# Beyond the Paddy Fields: Riparian Reed Beds as Essential Natural Habitat for the Critically Endangered Suweon Treefrog, *Dryophytes suweonensis*

**DOI:** 10.1002/ece3.74100

**Published:** 2026-07-30

**Authors:** Kyo Soung Koo, Eun Jin Park, Seung Nam Jin, Kwang Sung Yun

**Affiliations:** ^1^ Korean Environmental Geography Institute Sejong South Korea; ^2^ Division of EcoScience Ewha Womans University Seoul South Korea; ^3^ ECOnGEO Incheon South Korea

**Keywords:** anuran, East Asia, Hylidae, reed‐associated frog, riparian wetland, threatened species

## Abstract

The Suweon treefrog (*Dryophytes suweonensis* (Kuramoto, 1980)), a critically endangered amphibian endemic to the Korean Peninsula, is widely regarded as being closely associated with rice paddies. However, given that rice agriculture represents a relatively recent feature in the species' evolutionary history, strict dependence on this habitat warrants re‐examination. To assess habitat use during the non‐breeding season, we conducted intensive field surveys in Pyeongtaek, a core distribution area, during the pre‐breeding period of 2024. We detected 701 Suweon treefrogs, including 697 individuals within the 1.6 km^2^ study area (436 frogs/km^2^). No individuals were found inside rice paddies during the survey; instead, all were located on emergent vegetation within riparian waterways, with 97% occurring on the Common reed, *Phragmites australis* . These findings indicate that riparian reed habitats play a critical role during the non‐breeding season and may better reflect the species' ancestral ecological niche. Previous emphasis on rice paddies likely reflects a bias toward breeding‐season observations. Although rice paddies remain important breeding sites, conservation strategies focused solely on these habitats risk overlooking essential non‐breeding environments. We therefore recommend that conservation planning incorporate the protection and restoration of reed‐associated wetlands and that monitoring efforts encompass the full annual cycle. More broadly, our results highlight the need to reassess habitat associations in species traditionally considered dependent on anthropogenic environments.

## Graphical abstract

This figure shows the true habitat type of *Dryophytes suweonensis* that this paper aims to demonstrate.

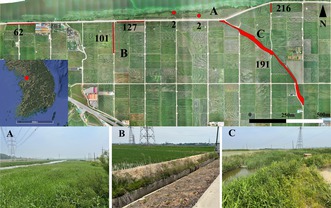

## 1 Introduction

Understanding habitat and space use by endangered species is fundamental to conservation biology, because management actions are typically directed at habitats rather than at individual organisms. These associations determine not only where species persist and reproduce, but also how they respond to anthropogenic change, habitat loss, and fragmentation (Fahrig 2003). Therefore, accurate knowledge of habitat use is essential for effective conservation planning (Guisan et al. 2013).

Reliable estimates of abundance and occupancy are equally crucial for conservation planning, yet they are often biased by methodological constraints, including surveys limited to a single habitat type or life‐history stage. For secretive or seasonally active species, counts based only on breeding sites or calling individuals may substantially underestimate population size and misrepresent habitat importance (MacKenzie et al. 2002; Pellet and Schmidt 2005; Mazerolle et al. 2007), with direct implications for protected‐area design and conservation priorities.

Agricultural landscapes illustrate these challenges particularly well, because rice agroecosystems and other man‐made wetlands simultaneously support high biodiversity and intense human use (Elphick 2000), creating complex trade‐offs between food production and wildlife conservation. Amphibians, in particular, often rely on artificial wetlands for breeding while utilizing alternative habitats for foraging and overwintering (Pope et al. 2000; Rittenhouse and Semlitsch 2007). Consequently, survey designs that focus solely on agricultural breeding sites may overlook critical non‐breeding habitats and mischaracterize species' ecological niches. The critically endangered Suweon treefrog ( *Dryophytes suweonensis* ) perfectly illustrates the dangers of such habitat mischaracterization.

The endangered Suweon treefrog was first identified in the Suwon area of Gyeonggi Province in 1976 by a specialist of East Asian *Hyla* (synonym *Dryophytes*) species (Kuramoto 1980). It was described as a new and endemic species under the name *D. suweonensis* , reflecting its type locality in Suwon (Kuramoto 1980; Song 2015; NIE 2019). The unique behavior of calling while perched and suspended among rice stems is a distinguishing characteristic of the Suweon treefrog (Kuramoto 1980; Borzée et al. 2016). In contrast to the widespread distribution of the Japanese treefrog ( *D. japonicus* ) distributed across the Korean Peninsula, the Suweon treefrog is restricted to a limited number of rice paddy areas along the west coast, and its population size is considered extremely small (Kim, Son, et al. 2012; Borzée and Jang 2017; Borzée, Andersen, et al. 2018; Do et al. 2021). The number of individuals observed rarely exceeds ten per locality (Roh et al. 2014; Borzée and Jang 2017; Borzée, Andersen, et al. 2018; Groffen et al. 2018). Consequently, in 2012, the Korean government classified the Suweon treefrog as threatened (EN B2b(iii–v)+C(iv)) and designated it as a Class I endangered wildlife species in order to protect and manage its population (NIE 2019). The International Union for Conservation of Nature (IUCN) has also assessed the Suweon treefrog as Endangered (EN) on its Red List (IUCN 2024a).

Rice paddies, cultivated in approximately 114 countries worldwide, represent one of the most widespread types of artificial wetlands (Kim et al. 2011; Muthayya et al. 2014; Propper et al. 2023). Despite their anthropogenic origin, they provide important habitats for a wide range of organisms, including invertebrates and various vertebrate taxa (Roger et al. 1991; Kiritani 2010; Propper et al. 2024). In South Korea, rice cultivation occupies approximately 15.7% of the national land area, and more than 100 species of invertebrates have been recorded in rice paddies (Cho et al. 2006). In addition, many Korean amphibian species, regardless of their primary habitats such as grasslands or forests, migrate to rice paddies for breeding (Do et al. 2021). The Suweon treefrog leaves rice paddies outside the breeding season and hibernates in their vicinity during winter, yet numerous studies indicate that the species is entirely dependent on rice paddies for survival during the breeding season (Borzée et al. 2015; Borzée and Jang 2019; Borzée 2020). These findings provide the foundation for current conservation and management strategies for the species (Borzée and Jang 2019; Park et al. 2021).

Previous studies on the Suweon treefrog have largely relied on auditory surveys of calling males in rice paddies during the breeding season (Borzée, Heo, et al. 2018). While such methods are widely used for anuran monitoring, they can underrepresent non‐calling individuals, other life stages, and individuals occupying non‐paddy habitats, thereby biasing both abundance estimates and inferences about habitat importance (Weir et al. 2005; Lotz and Allen 2007). This raises the possibility that the species' true population size and its reliance on non‐paddy habitats have been underestimated, underscoring the need for survey designs that encompass multiple habitat types and activity periods. While visual encounter surveys can complement acoustic monitoring by detecting non‐calling individuals, they may also introduce their own detection biases, for example if some habitats, such as reed beds, facilitate easier observation than others. For species that use different habitat types across their annual cycle, breeding‐season surveys can be particularly misleading, because individuals aggregate at reproductive sites that may not reflect their primary non‐breeding habitat (Rittenhouse and Semlitsch 2007). In amphibians, this can create the impression that artificial breeding sites such as rice paddies constitute the main habitat, even when individuals spend most of their active season in alternative environments (Natuhara et al. 2012; Borzée, Choi, et al. 2018). To avoid this bias and better characterize non‐breeding habitat use, it is necessary to survey individuals before they fully concentrate in breeding habitats and to include adjacent habitat types such as riparian vegetation and irrigation channels in sampling designs (Dickerson and Oliver 2001). In the case of the Suweon treefrog, focusing on the pre‐breeding period allows us to test whether reed‐dominated riparian habitats function as key natural environments outside the short breeding season in rice paddies (Roh et al. 2014; Song 2015; Do et al. 2017; Ryu and Lee 2018; Lee et al. 2019; Do et al. 2021; Koo et al. 2022). Although a few studies have noted Suweon treefrogs in irrigation channels or semi‐natural wetlands, none have provided quantitative data on microhabitat use within reed‐dominated waterways outside the breeding season.

Meanwhile, although the exact origin of rice cultivation in Korea remains unclear, radiocarbon dating of rice grains discovered at archaeological sites suggests that it began approximately 2000–4000 years ago (e.g., Lee et al. 1994; Kim 2015). In contrast, phylogenetic and biogeographic studies of Holarctic *Dryophytes* treefrogs indicate that diversification within this clade, including lineages closely related to the Suweon treefrog, occurred on much longer timescales (on the order of tens of thousands to millions of years), implying that the species is unlikely to have evolved exclusively in rice paddies (Li et al. 2015; Borzée, Didinger, et al. 2017; Shimada et al. 2025). This indicates that rice paddies—the only currently documented breeding habitat of the Suweon treefrog—are relatively recent anthropogenic environments on the Korean Peninsula. While this does not allow us to infer evolutionary origin, it suggests that other, more natural wetland habitats may historically have played an important role in its ecology.

In June 2021, multiple individuals of Suweon treefrogs were reported from irrigation channels near rice paddies in the Paju region (Kim et al. 2021). Notably, they were observed not in the rice paddies themselves, but in adjacent irrigation channels and on surrounding plants, leading previous researchers to suggest that these structures and associated vegetation may serve as important habitats for the species (Borzée 2024). In our study area, extensive stands of Common reed ( *Phragmites australis* ) line these irrigation channels and riverbanks, forming dense emergent vegetation typical of riparian wetlands. Building upon these preliminary observations, we formally tested the hypothesis that riparian reed beds constitute a key non‐breeding habitat for the Suweon treefrog, potentially approximating its ancestral, pre‐agricultural environment more closely than modern rice paddies. To test this hypothesis, we analyzed microhabitats such as rice paddies, plants, and artificial structures within and around areas occupied by the Suweon treefrog. This study aimed to identify the key natural habitat types currently used by the critically endangered Suweon treefrog during the non‐breeding season, thereby providing a crucial scientific basis for its effective conservation and management. Furthermore, our findings may prompt a broader reconsideration of whether rice paddies truly represent the primary habitat for species currently considered dependent on agroecosystems.

## 2 Materials and Methods

### 2.1 Study Area

The critically endangered Suweon treefrog has been documented as occurring only in a limited number of rice paddies near major rivers extending inland from the western coast and estuarine regions of the Korean Peninsula (Do et al. 2017; Koo and Choe 2021; Borzée et al. 2021). We reviewed previous studies to identify regions where the Suweon treefrog has been repeatedly recorded in the literature. We selected a site in Pyeongtaek (N37 00, E126 50) where the presence of the Suweon treefrog has been consistently documented as the study area; detailed coordinates are withheld to protect the species. Although the Suweon treefrog population in this area was reported to be nearly extinct in 2014 (IUCN SSC Amphibian Specialist Group 2024b), recent studies have documented the continued presence of numerous individuals at this site (Do et al. 2021; Koo and Choe 2021). The rice paddy area at the study site covers approximately 1.6 km^2^ and contains a network of irrigation channels of varying sizes. Adjacent to the study site are Asan Lake and Namyang Lake, both of which were formed by damming rivers that flow into the West Sea.

### 2.2 Field Survey and Data Analysis

The Suweon treefrog typically begins its main breeding activities in late May to early June, following the flooding of rice paddies and transplanting of rice seedlings (Kim, Ham, et al. 2012). To determine the onset of activity in the Suweon treefrog, we visited the study site one to two times per week from April 29, 2024, monitoring for evidence of activity. On the night of May 20, the Suweon treefrog was first observed moving along the rice paddy bund. Beginning the following day, May 21, we initiated surveys of habitat types and microenvironmental conditions, focusing on rice paddies, waterways, and adjacent vegetation, guided by previous reports of Suweon treefrogs occurring near rice paddies and irrigation channels. Field surveys were conducted from 08:00 to 24:00, encompassing both daytime and nighttime periods. On May 21, when the field survey commenced, the rice paddies had been flooded in preparation for planting, but by May 26, rice seedlings had begun to be transplanted into the fields. For hypothesis testing in this study, it was necessary to identify whether Suweon treefrogs predominantly use reed‐dominated riparian habitats, rather than rice paddies, prior to their full breeding activity. We therefore focused our surveys on the pre‐breeding period, when individuals are active but have not yet aggregated in paddies, allowing us to capture non‐breeding habitat use in surrounding riverine and reed‐dominated environments. Field surveys were conducted for four consecutive days, until May 25, before rice seedlings were transplanted into the paddies.

In the study area, all rice paddy plots are separated by narrow bunds and access roads, and we surveyed the entire system by walking along every bund and road between paddies. To minimize double counting of individuals, we did not repeatedly traverse the same bunds or roads within a short time interval, and each frog was recorded only once per survey, based on its location, observation time, and photographs. All observed individuals were photographed using a digital camera (SX70 HS, Canon, Japan). The approximate position of each frog was recorded in the field by marking its location directly on high‐resolution satellite imagery in Google Earth using a smartphone, with coordinates stored in WGS84 geographic format (latitude/longitude). To ensure consistency, we visually matched each marked point to the corresponding irrigation channels and reed stands visible on the imagery and used these coordinates for subsequent mapping and density calculations.

Surveys were conducted using a non‐invasive protocol without capturing or handling frogs. All individuals were recorded visually and photographed, but we did not attempt to mark or identify individuals from photographs, as this was not feasible given the large sample size and similar external appearance. The observation distance was maintained at approximately 3 m to avoid disturbing frogs, and no obvious behavioral responses (e.g., flight or cessation of activity) were observed during surveys. Search effort was standardized by visually scanning all accessible microhabitats along bunds and access roads, including all emergent vegetation within irrigation channels and adjacent reed beds, rather than restricting effort to particular habitat types.

The distribution of the Suweon treefrog is restricted to the western region of the Korean Peninsula, including some inland areas along river systems, and overlaps throughout much of its range with that of its closely related species, the Japanese treefrog (Borzée, Kim, et al. 2017; Do et al. 2017, 2021). Although the two species are morphologically similar, we distinguished them using a validated identification key based on body shape, coloration, and the recently identified dorsal pattern and tympanum morphology of the Suweon treefrog (Kim et al. 2021; Park 2026).

All plant species associated with the occurrence of Suweon treefrogs were recorded, and species identification was based on the literature of Lee (2003), Kim (2016), and Cho et al. (2016). For alien plant species, the criteria of Park (2009) and Kim and Kil (2017) were followed. In addition, the positions of Suweon treefrogs on plants were classified as either “leaf” or “stem”. The height at which individuals were observed was not measured in this study, because entering the habitat to make such measurements could have caused disturbance.

**FIGURE 1:**
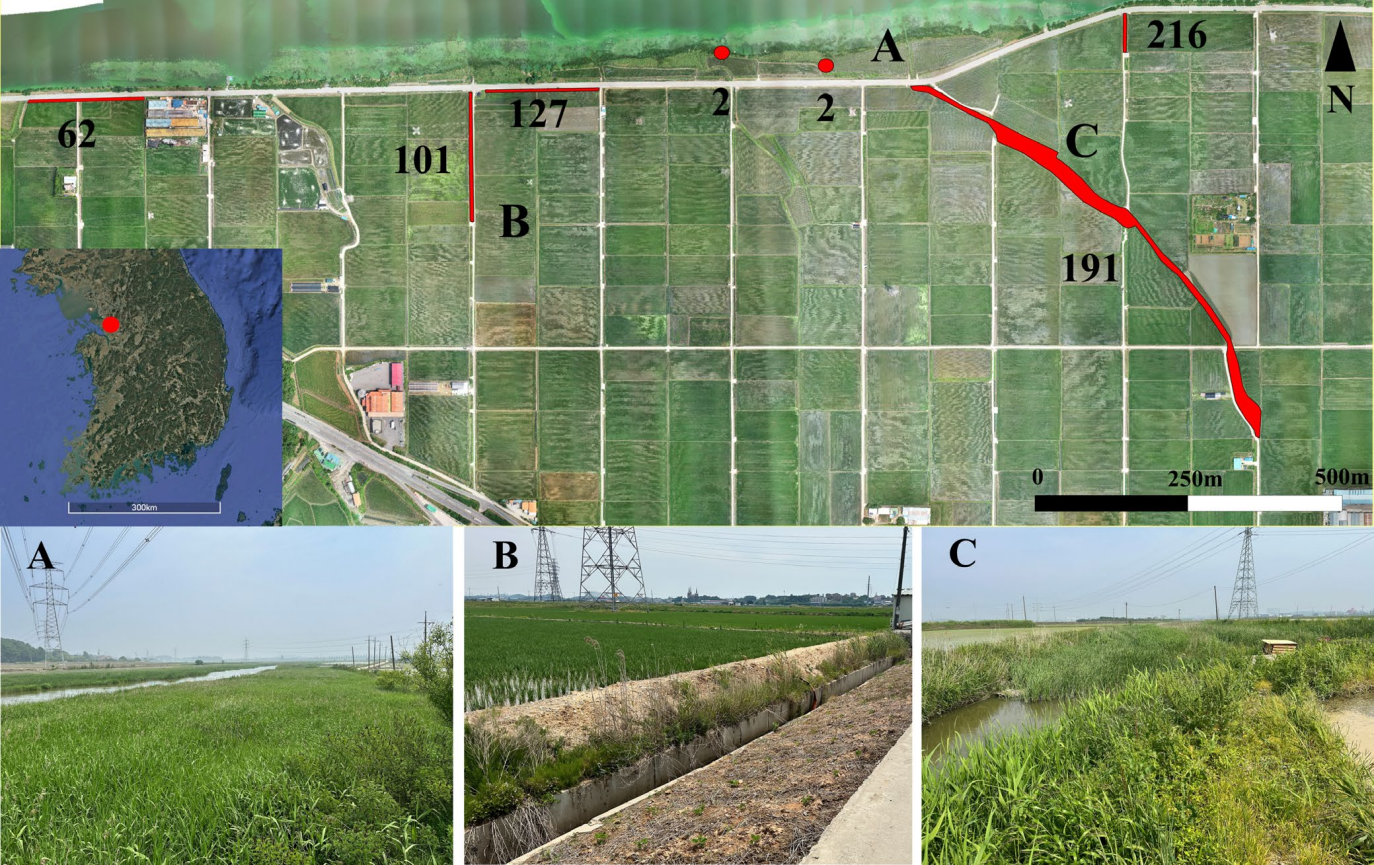
Distribution of the Suweon treefrog ( *Dryophytes suweonensis* ) in Pyeongtaek, South Korea. Red dots and polygons indicate locations where Suweon treefrogs were observed, with numbers representing the number of individuals. (A) reed beds, (B) concrete‐lined canal, (C) semi‐natural canal.

Population density was calculated as the total number of observed individuals divided by the effective survey area (1.6 km^2^), defined as the mapped polygon encompassing rice paddies, irrigation channels, and adjoining reed beds that were systematically surveyed (Figure 1). Unsuitable habitats, such as buildings and non‐surveyed forest patches, were excluded. For the four individuals found in the reed community of the estuarine zone (indicated as two red dots in Figure 1), the precise habitat area could not be estimated; therefore, these records were excluded from the density analyses.

Plant species used by Suweon treefrogs were classified at the order, family, genus, and species levels. The differences in the number of frogs among specific habitat sections and the frequencies at each taxonomic level were compared using chi‐square tests. Differences in the frequencies of positions on the recorded plants were also analyzed using a chi‐square test. We used chi‐square goodness of fit tests to assess whether the observed numbers of frogs differed among plant species and habitat sections. Expected frequencies were set equal across categories because we did not quantify habitat availability (e.g., proportional area or cover of each plant species), and we checked that expected counts were above commonly recommended thresholds. These tests therefore indicate unequal use among the available categories, but are not formal resource‐selection models comparing use with availability. Statistical significance was determined at *p* < 0.05. Statistical analyses were performed using the SPSS software program (v26.0, IBM, USA).

### 2.3 Research Permit

This study was conducted with the approval of the Institutional Animal Care and Use Committee of Ewha Womans University (EWHA IACUC 24‐027). According to the Korean Wildlife Protection and Management Act, the capture of endangered species—including the Suweon treefrog (classified as Endangered Wildlife Class I)—is strictly prohibited. However, permission may be granted for purposes such as research (Ministry of Government Legislation 2024). In this study, a non‐invasive approach was employed in which the locations of Suweon treefrog individuals were confirmed and recorded solely through visual observation and photography, without direct capture. Therefore, no capture or animal research ethics permit was required for the Suweon treefrogs.

## 3 Results

**FIGURE 2:**
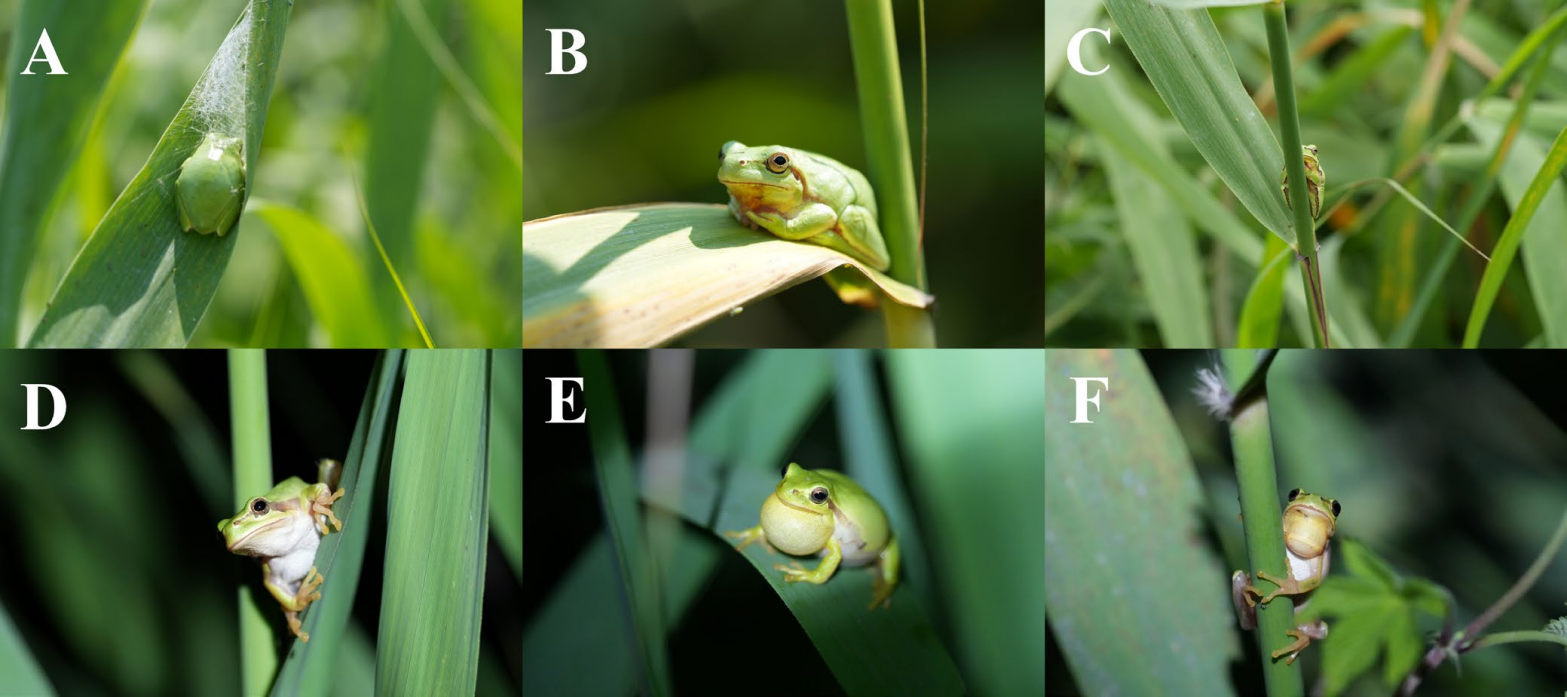
The Suweon treefrogs ( *Dryophytes suweonensis* ) observed on the stems and leaves of Common reed ( *Phragmites australis* ). (A–C) Individuals resting on reeds during the day; (D) a female foraging at night; (E, F) calling males on reeds.

During the survey period, a total of 701 individuals were; however, population density was calculated based only on the 697 individuals occurring within the 1.6 km^2^, corresponding to a population density of 436 individuals/km^2^ (Figure 1). The Suweon treefrog was not observed in buildings, forests, or rice paddies, but was found exclusively on plants within waterways. A total of 31 plant species (12 orders and 14 families) were identified in the study area (Table 1). Additional data on plant species used by Suweon treefrogs and the specific plant parts (leaf vs. stem) occupied by individuals are presented in Supporting Information. Specifically, 97.3% (*n* = 682) of individuals were found on Poaceae (order Poales), while only 2.7% (*n* = 19) were observed on Polygonaceae (order Caryophyllales) (*χ* ^2^ = 627.06, df = 1, *p* < 0.001). At the species level (Figure 2), the Suweon treefrogs were most frequently observed on Common reed (97.0%, *n* = 680), followed by Curly dock (2.7%, *n* = 19), Runner reed (0.1%, *n* = 1), and Manchurian wild rice (0.1%, *n* = 1) (*χ* ^2^ = 1939.59, df = 3, *p* < 0.001). Although the Suweon treefrogs were found on four different plant species, all individuals were observed on plants co‐occurring within stands of the common reed, *P. australis* . In addition, 93.2% (*n* = 653) of the Suweon treefrogs were found on leaves, while only 6.8% (*n* = 48) were observed on stems (*χ* ^2^ = 522.15, df = 1, *p* < 0.001).

**TABLE 1:** Plant species identified in the Suweon treefrog habitats and their frequencies of utilization.

Order	Family	Scientific name	English name (Korean)	*N* (%)
Apiales	Apiaceae	*Oenanthe javanica*	Chinese celery (미나리)	
Asterales	Asteraceae	*Artemisia indica*	Korean wormwood (쑥)	
		*Hemistepta lyrata*	Hemistepta (지칭개)	
		*Lactuca serriola*	Prickly lettuce (가시상추)	
		*Erigeron annuus*	Annual fleabane (개망초)	
		*Sonchus asper*	Prickly sow‐thistle (큰방가지똥)	
		*Youngia japonica*	Oriental false hawksbeard (뽀리뱅이)	
Caryophyllidae	Amaranthaceae	*Achyranthes bidentata*	Ox knee (쇠무릎)	
Poales	Typhaceae	*Typha angustifolia*	Lesser bulrush (애기부들)	
	Poaceae	*Bromus tectorum*	Downy brome (털빕새귀리)	
		*Elymus tsukushiensis*	Wheatgrass (개밀)	
		*Festuca arundinacea*	Tall fescue (큰김의털)	
		*Phragmites australis*	Common reed (갈대)	680 (97.0)
		*Phragmites japonica*	Runner reed (달뿌리풀)	1 (0.1)
		*Setaria viridis*	Green foxtail (강아지풀)	
		*Zizania latifolia*	Manchurian wild rice (줄)	1 (0.1)
Equisetales	Equisetaceae	*Equisetum arvense*	Common horsetail (쇠뜨기)	
		*Equisetum ramosissimum*	Branched horsetail (개속새)	
Fabales	Fabaceae	*Amorpha fruticose*	Desert false indigo (족재비싸리)	
		*Glycine soja*	Wild soybean (돌콩)	
		*Lespedeza cuneat*	Chinese bushclover (비수리)	
		*Vicia sativa*	Common vetch (살갈퀴)	
		*Vicia sepium*	Bush vetch (구주갈퀴덩굴)	
Ranunculales	Ranunculaceae	*Ranunculus tachiroei*	Tachiroei buttercup (개구리미나리)	
Malpighiales	Salicaceae	*Salix pierotii*	Korean willow (버드나무)	
Caryophyllales	Polygonaceae	*Persicaria thunbergii*	Thunberg's smartweed (고마리)	
		*Rumex crispus*	Curly dock (소리쟁이)	19 (2.7)
Rosales	Cannabaceae	*Humulus japonicus*	Japanese Hop (환삼덩굴)	
	Rosaceae	*Rubus parvifolius*	Japanese bramble (멍석딸기)	
Gentianales	Rubiaceae	*Galium spurium*	Stickwilly (갈퀴덩굴)	
Solanales	Convolvuaceae	*Calystegia sepium*	Hedge bindweed (큰메꽃)	

*Note:* Percentages may not sum to 100% due to rounding.

## 4 Discussion

The main finding of this study is that a large number of Suweon treefrogs were observed along river edges, in irrigation channels, and in the associated vegetation surrounding rice paddies prior to their agricultural use. These results suggest that reed beds are likely closer to the species’ natural non‐breeding habitats than rice paddies, indicating that future research and conservation efforts should also explicitly consider riparian reed beds. Our data describe habitat use during a short pre‐breeding period at a single locality, so we do not infer evolutionary origin, overwintering ecology, or full annual habitat dependence from these observations. Because this study is confined to a specific region and time period, further research is needed to clarify the broader distribution of the Suweon treefrog and the ecological roles of each habitat type. Nevertheless, without a shift in monitoring and conservation strategies to include non‐rice paddy habitats, the recovery of the Suweon treefrog may become increasingly difficult. Although our results indicate a strong association between the species and reed habitats, we did not formally estimate detection probabilities across habitat types; therefore, we interpret this pattern as evidence of association rather than as definitive absence in non‐reed habitats.

### 4.1 Rice Paddy Serves as a Functional Habitat for Breeding

Rice paddies have long been regarded as the main habitat for the endangered Suweon treefrog, serving as sites for breeding, foraging, and hibernation. As a result, most ecological and conservation studies, as well as management strategies, have focused almost exclusively on rice paddy environments (Borzée et al. 2015; Borzée and Jang 2017, 2019; Borzée, Andersen, et al. 2018; Borzée, Choi, et al. 2018; Borzée, Heo, et al. 2018; Borzée, Kim, et al. 2018; Groffen et al. 2018; Borzée 2020). However, the present study demonstrates that reed beds and their associated habitats are important environments utilized by this species, complementing rice paddies as key parts of its habitat use. Rice paddies have long been regarded as the main habitat for the endangered Suweon treefrog, serving as sites for breeding, foraging, and hibernation. Given that speciation within *Dryophytes* occurs over much longer timescales than the 2000–4000 years since the emergence of rice agriculture, it seems unlikely that rice paddies alone represent the environment in which the Suweon treefrog evolved (Lee et al. 1994; Ahn 2010; Shimada et al. 2025). However, species can rapidly adjust to human‐modified habitats, and our argument is intended to highlight the plausibility of alternative natural wetland habitats, such as riparian reed beds, rather than to provide definitive evolutionary evidence. Despite this, the species was first discovered in rice paddies (Kuramoto 1980), which may have led researchers to focus on these habitats and overlook the potential importance of riverine and reed habitats. Furthermore, amphibians inhabiting riparian vegetation have not been documented in South Korea, contributing to this oversight. This focus on rice paddies may, in part, explain the limited conservation success for the Suweon treefrog despite significant investment (Lee 2019; Jang 2020; IUCN SSC Amphibian Specialist Group 2024b). Nonetheless, rice paddies remain important contemporary breeding sites for the species. Therefore, for effective conservation, it is important to expand protection efforts to include not only rice paddies but also adjacent waterways and vegetation.

### 4.2 Ecological Role of Reed Beds for the Suweon Treefrog

Common reed is a representative emergent macrophyte that forms colonies and is distributed primarily in river estuaries. It is a dominant species among coastal plants in South Korea, accounting for approximately 62.7% of the total coastal vegetation (Rho 2007; Jin 2017). The distribution of reed beds is closely associated with the presence of rice paddies near rivers and estuaries (Ryu and Lee 2018; Lee et al. 2019). As a result, populations of the Suweon treefrog that formerly inhabited these riparian reed beds are now restricted to a few remaining areas that have persisted after large‐scale conversion of such habitats into rice paddies (Song 2015). Despite this, the continued survival of the Suweon treefrog likely depends not only on the remaining reed beds but also on rice paddies, which serve as breeding habitats from May to July (Kim, Ham, et al. 2012). In fact, the frequent occurrence of the Suweon treefrog in the lower reaches of the Gongneung Stream in Paju and nearby rice paddies suggests that reed beds remain important habitats for the species (Kim, Son, et al. 2012; Han 2013; Song 2015).

The ecological background for the predominant occurrence of the Suweon treefrog in reed beds may be influenced by historical shifts in distribution, whereby the species migrated upstream following these reed beds as sea levels rose after the last glacial period (Yang 2008; Borzée et al. 2020). Reed beds are highly prevalent in rivers and estuaries, and their dominance over other plant species supports the argument that they provide key natural habitat for the Suweon treefrog in riverine and estuarine landscapes (Lee et al. 2008; Kong et al. 2014; Jin 2017). In contrast, the Suweon treefrogs are rarely observed in the Runner reed (*P. japonica*), another species in the same genus. This likely results from the distinct ecological niche of, which is found mainly in upstream and mountainous areas (Lee 2008). If the Suweon treefrogs also preferred the Runner reed, their distribution would likely be broader and include more inland and forested areas. Thus, the current habitat range of the Suweon treefrog appears to be shaped by its association with reed beds.

Reed beds (=*Phragmites* stands) provide various ecological benefits, including moisture retention, water purification, shading, and predator avoidance. In particular, the structure of *Phragmites* leaves and stems offers a stable environment for the Suweon treefrogs to rest and avoid predation (Kong et al. 2014; Park and Lee 2018). These characteristics help explain why the species is frequently associated with reed beds and further support the hypothesis that these habitats may better reflect the species' ecological requirements than rice paddies. It is important to note that our study did not quantify habitat availability, such as the proportional area or cover of reeds versus alternative vegetation types, along the surveyed waterways. Because frog observations were spatially clustered along certain canal segments, we did not attempt to correct for spatial autocorrelation. Our chi‐square analyses therefore describe differences in usage among plant species, but they do not constitute a formal habitat‐selection analysis based on use versus availability. We therefore interpret the high proportion of observations on *Phragmites* as evidence of a strong association with reed‐dominated habitats at this locality, while acknowledging that true preference and specialization would need to be evaluated using resource‐selection or occupancy modeling frameworks. Future studies that quantify the relative availability of different vegetation types and apply resource selection functions or occupancy models across multiple sites and seasons will be essential to formally test habitat preferences and potential specialization in the Suweon treefrog. We did not quantify environmental covariates such as water depth, temperature, humidity, vegetation density, predator presence, or prey abundance within the surveyed waterways. However, all plant species used by frogs occurred within the same irrigated canal and riparian system, where such conditions are expected to be broadly similar. Our discussion of potential mechanisms, such as moisture retention, structural support, and predator avoidance, should therefore be regarded as plausible ecological hypotheses rather than demonstrated causal relationships. Going forward, investigating the distribution of reed beds along brackish and riverine zones and its relationship with the presence of Suweon treefrogs will be crucial for understanding the habitat requirements and range of this endangered species.

### 4.3 Reed‐Associated Ecology of the Suweon Treefrog

Frogs of the family Hyperoliidae, commonly known as reed frogs, primarily inhabit southern Africa and thrive in environments dominated by tall grasses, sedges, and reed beds (von Rapp 1842). These habitats, characterized by elongated and slender vegetation, support key biological activities such as foraging, breeding, and resting (Largen 1998). Although this similarity does not imply any phylogenetic or biogeographic relationship between the Suweon treefrog and African reed frogs, the ecological adaptations of members of the Hyperoliidae may still offer valuable analogs for interpreting the habitat specialization of the Suweon treefrog. In the present study, most Suweon treefrogs were observed in reed beds rather than rice paddies, which have traditionally been considered their primary habitat. Notably, individuals were absent from trees or shrubs, habitats typical of treefrogs. This preference suggests that the Suweon treefrogs may be more ecologically similar to reed‐dwelling frogs, with traits adapted to reed‐dominated rather than arboreal environments. Its slender body form (Kuramoto 1980; Borzée et al. 2013) and long, longitudinal dorsal stripes (Kim et al. 2021) further support specialization for movement within reed beds rather than tree climbing (Schiøtz 1999). Moreover, closely related species such as the Spotless treefrog ( *D. immaculatus* ), which shares approximately 99% genetic similarity in mtDNA, may display some parallel ecological and morphological features (Li et al. 2015; Borzée, Didinger, et al. 2017; Lee et al. 2017; Shimada et al. 2025). These patterns collectively underscore a strong association between the Suweon treefrog and reed‐dominated habitats and support the hypothesis that its ecology is closely tied to reed beds.

### 4.4 Limitations of Previous Research Methods and Underestimation for the Species

Across most recorded areas where the Suweon treefrog has been observed to date, typically fewer than 20 individuals have been documented (Roh et al. 2014; Borzée and Jang 2017; Borzée, Andersen, et al. 2018; Groffen et al. 2018). The largest population identified so far was in Gimpo, with 31 individuals recorded in a single paddy patch in 2019 (Koo et al. 2022). In contrast, in this study, over 700 individuals were recorded at a single locality, and nearly 200 were found within a watercourse approximately 70 m in length. These estimates are likely conservative, as population counts were made from outside the habitat to minimize disturbance, suggesting that the actual number may be even higher. Earlier studies primarily concentrated on calling individuals detected in rice paddies (see the studies by Borzée). Consequently, previous counts were often limited to males producing advertisement calls in breeding sites, significantly underestimating the true population size. Moreover, subadults, females, and non‐calling or non‐breeding males—such as satellite males—may have been excluded from estimates (Bae et al. 2024).

Most previous studies have relied on these limited survey methodologies, resulting in conservation measures developed from potentially skewed data. For example, Borzée, Andersen, et al. (2018) estimated population size solely by counting advertisement calls, but the resulting estimates varied greatly among sites. Such limited survey approaches likely resulted in a significant underestimation of actual population sizes, rendering observed numbers highly susceptible to fluctuations caused by small sample sizes. These apparent variabilities may have also contributed to misinterpretations regarding the decline of the Suweon treefrog (Borzée, Andersen, et al. 2018). Moreover, since the number of breeding males can vary with season or time of day, conducting sequential auditory monitoring across multiple sites may introduce further bias (Kim et al. 2012). Hence, alongside acoustic monitoring, direct counts of individuals in these newly identified habitat types are necessary to more accurately assess the population structure and abundance of this endangered species. While our pre‐breeding surveys help to reveal non‐breeding habitat use that may be under‐represented in breeding‐season, paddy‐focused studies, they do not cover the full annual activity period, and thus represent a different but complementary seasonal perspective rather than a complete correction of previous biases. At the same time, we acknowledge that our visual counts may also be affected by habitat‐specific detectability, and that formal occupancy‐detection models will be needed to separate true habitat use from differences in detection probability among vegetation types.

### 4.5 The Largest Habitat and the Necessity of Habitat Conservation

In the study area, the density of the Suweon treefrogs exceeded 400 individuals per square kilometer, and both the absolute population size and density recorded here surpass all previously reported localities (Roh et al. 2014; Borzée, Didinger, et al. 2017; Borzée, Andersen, et al. 2018; Groffen et al. 2018; Koo et al. 2022). This strong association with reed‐dominated habitats and high local density suggests that the Suweon treefrogs may be particularly vulnerable to disturbances such as habitat loss and pollution. Riverbanks and brackish zones that support large aggregations of the Suweon treefrogs are under intense development pressure in South Korea (Ryu and Lee 2018). Between 2008 and 2016, sandbar area along the four largest rivers declined by approximately 95% (from 15.2 to 0.7 km^2^), while riparian zones decreased by nearly half (from 56.1 to 28.9 km^2^) (Ban 2021). Similarly, rice paddies—critical breeding sites for the Suweon treefrogs—shrunk by 64.3%, from 2.32 million hectares in 1968 to 0.829 million hectares in 2011 (Chae 2012). Given these dramatic habitat losses and ongoing threats, there is an urgent need to accurately assess the current distribution and population size of the Suweon treefrog and to develop and implement effective, habitat‐inclusive conservation strategies.

### 4.6 Proposed Methods for Effective Monitoring for This Species

Previous studies on the Suweon treefrog have predominantly relied on auditory surveys in rice paddies, which likely introduced biases by overlooking individuals inhabiting non‐paddy environments (see studies by Borzée). The present study demonstrates that substantial numbers can also be found in riverine and reed‐dominated habitats. To improve future monitoring, we propose several methodological enhancements. First, the temporal scope should be broadened beyond the traditional breeding season (May–July) to include both pre‐ and post‐breeding active periods (March–April and August–November), reflecting observed extended activity into late autumn (Park 2026). Since outside the brief breeding season, a significant proportion of the population may rely on alternative habitats such as reed beds and adjacent riparian vegetation, surveys focusing solely on paddies risk missing large segments of the population. Second, incorporating diurnal visual encounter surveys alongside nocturnal auditory methods will help address previous biases toward adult calling males and provide a more comprehensive population assessment. Collectively, these improvements will enable more accurate and effective long‐term monitoring of this endangered species. To move beyond descriptive patterns, future work should incorporate more formal habitat‐selection and spatial modeling approaches, including (i) analyses that account for habitat availability and spatial auto‐correlation, (ii) estimation of confidence intervals and effect sizes for habitat associations, (iii) occupancy‐detection models that jointly estimate habitat use and detection probability, and (iv) multivariate analyses of environmental covariates such as vegetation structure, water depth, and microclimate. Our present study is best viewed as a detailed descriptive baseline that identifies key non‐breeding habitats and motivates these more complex analyses, while providing a new perspective on habitat use in the Suweon treefrog and prompting a critical re‐evaluation of conservation strategies that have so far focused almost exclusively on rice paddies.

## 5 Conclusion

The present study suggests that the prevailing view that rice paddies constitute the primary habitat of the Suweon treefrog should be revised, because the species extensively uses reed‐dominated riparian habitats outside the breeding season. Our results suggest that the long‐standing perception of the species as a rice paddy specialist may, at least in part, reflect a historical emphasis on breeding‐season surveys, and that the ecological importance of non‐paddy habitats, as well as the overall population size of the species, may have been underestimated. Because both rice paddies and riparian wetlands in South Korea are undergoing rapid change due to land development and agricultural intensification, effective conservation planning should consider habitats used throughout the species' annual cycle rather than focusing solely on breeding sites. In particular, future monitoring and management programs would benefit from explicitly incorporating reed‐dominated wetlands and adjacent waterways, and from applying both nocturnal and diurnal surveys across the full activity season to capture the species' broader habitat use. Additional ecological studies across the species' distribution are required to evaluate whether the habitat‐use patterns documented here are representative of its range‐wide ecology and to refine habitat‐based conservation strategies. A more complete understanding of the habitat spectrum used by the Suweon treefrog will provide a stronger basis for the long‐term conservation of this endangered species.

## Author Contributions

**Kyo Soung Koo:** conceptualization (lead), data curation (lead), investigation (equal), methodology (equal), supervision (lead), writing – original draft (lead), writing – review and editing (equal). **Eun Jin Park:** investigation (equal), visualization (lead), writing – review and editing (equal). **Seung Nam Jin:** investigation (equal), methodology (equal), writing – review and editing (equal). **Kwang Sung Yun:** funding acquisition (equal), methodology (equal), resources (equal), writing – review and editing (equal).

## Funding

This work was supported by the Ministry of Education (Grant RS‐2024‐00392032).

## Conflicts of Interest

The authors declare no conflicts of interest.

## Supporting information


**Data S1:** ece374100‐sup‐0001‐Supinfo.xlsx.

## Data Availability

The data that supports the findings of this study are available in the Supporting Information of this article.
